# Comprehensive evaluation of plasma microbial cell-free DNA sequencing for predicting bloodstream and local infections in clinical practice: a multicenter retrospective study

**DOI:** 10.3389/fcimb.2023.1256099

**Published:** 2024-01-04

**Authors:** Feng Pang, Wenbin Xu, Hui Zhao, Shuai Chen, Yaxian Tian, Juanjuan Fu, Zhiqing You, Pingping Song, Qingjie Xian, Qigang Zhao, Chengtan Wang, Xiuqin Jia

**Affiliations:** ^1^ Department of Clinical Laboratory, Liaocheng People’s Hospital, Liaocheng, Shandong, China; ^2^ Department of Clinical Laboratory, Liaocheng Thrid People’s Hospital, Liaocheng, Shandong, China; ^3^ Department of Center Laboratory, Liaocheng People’s Hospital, Liaocheng, Shandong, China; ^4^ The Key Laboratory of Molecular Pharmacology, Liaocheng People’s Hospital, Liaocheng, Shandong, China

**Keywords:** metagenomic next-generation sequencing (mNGS), plasma cell-free DNA (cfDNA), bloodstream infections, local infections, ROC curves

## Abstract

**Background:**

Metagenomic next-generation sequencing (mNGS) of plasma cell-free DNA (cfDNA) shows promising application for complicated infections that cannot be resolved by conventional microbiological tests (CMTs). The criteria for cfDNA sequencing are currently in need of agreement and standardization.

**Methods:**

We performed a retrospective cohort observation of 653 patients who underwent plasma cfDNA mNGS, including 431 with suspected bloodstream infections (BSI) and 222 with other suspected systemic infections. Plasma mNGS and CMTs were performed simultaneously in clinical practice. The diagnostic efficacy of plasma mNGS and CMTs in the diagnosis of blood-borne and other systemic infections was evaluated using receiver operating characteristic (ROC) curves. The sensitivity and specificity of the two methods were analyzed based on the final clinical outcome as the gold standard.

**Results:**

The mNGS test showed an overall positive rate of 72.3% (472/653) for detecting microorganisms in plasma cfDNA, with a range of 2 to 6 different microorganisms detected in 171 patient specimens. Patients with positive mNGS results were more immunocompromised and had a higher incidence of severe disease (P<0·05). The sensitivity of mNGS was higher for BSI (93·5%) and other systemic infections (83·6%) compared to CMTs (37·7% and 14·3%, respectively). The mNGS detected DNA from a total of 735 microorganisms, with the number of microbial DNA reads ranging from 3 to 57,969, and a higher number of reads being associated with clinical infections (P<0·05). Of the 472 patients with positive mNGS results, clinical management was positively affected in 203 (43%) cases. Negative mNGS results led to a modified clinical management regimen in 92 patients (14.1%). The study also developed a bacterial and fungal library for plasma mNGS and obtained comparisons of turnaround times and detailed processing procedures for rare pathogens.

**Conclusion:**

Our study evaluates the clinical use and analytic approaches of mNGS in predicting bloodstream and local infections in clinical practice. Our results suggest that mNGS has higher positive predictive values (PPVs) for BSI and systemic infections compared to CMTs, and can positively affect clinical management in a significant number of patients. The standardized whole-process management procedure for plasma mNGS developed in this study will ensure improved pre-screening probabilities and yield clinically valuable data.

## Introduction

Bloodstream infections (BSI) are infectious diseases that may endanger the lives of critically ill patients (Rhee et al., 2019) and traditional blood culture methods are the gold standard for diagnosing BSI (Rhodes et al., 2017; Miller et al., 2018). However, blood culture methods can only identify the pathogen in less than 20% of sepsis cases (Miller et al., 2018); most pathogens are not detected in more than half of patients in routine clinical testing (Blauwkamp et al., 2019). In recent years, nucleic acid molecular diagnostic techniques have been increasingly used to directly test blood cultures as a means to improve diagnostic accuracy and accelerate pathogen identification (Marschal et al., 2017). Molecular assays need to predict the presence of possible infectious pathogens in advance and are designed to detect specific targets, which increases the detection bias. These techniques have noticeable deficiencies in clinical practice, especially for rare unknown pathogens, where identification is confirmed with positive blood culture. Metagenomic next-generation sequencing (mNGS) is an advanced technology applied to pathogen diagnostic practice in recent years. It can detect pathogens’ genomes in clinical samples without relying on culture enrichment and amplification (Greninger et al., 2017; Chen et al., 2020). Additionally, mNGS allows detection and identification of a virtually unlimited range of organisms in a single test without prior selection of target pathogens (Gu et al., 2019). In clinical practice, the detection of plasma cell-free DNA mNGS is not commonly used to examine BSI (Carbo et al., 2022). A study by Blauwkamp et al. (2019) showed that the sensitivity of mNGS for sepsis diagnosis is much higher than that of all other methods.

Because blood flows through various tissues and organs throughout the body, even localized infections outside the blood system may have enough pathogenic DNA fragments to enter the bloodstream to be detected by mNGS (Farnaes et al., 2019). A previous study showed that fragments of genomic DNA from pathogens infecting other parts of the body can be detected in purified plasma cell-free DNA (cfDNA) (Song et al., 2021), suggesting the possibility of noninvasive detection of various infections by sequencing plasma microbial cfDNA. Plasma mNGS has many advantages over conventional microbiological tests (CMTs), the most important being able to detect microbial cfDNA fragments in the plasma of patients without BSI. These fragments may be related to commensal microorganisms irrelevant to sepsis or incidentally discovered. However, it’s not possible to distinguish whether the detected cfDNA fragments originate from the responsible infectious pathogen or from transient bacteremia caused by the colonizing bacteria. Clinicians and laboratories alike are facing the challenge of determining the optimal utility of mNGS technology. This challenge arises from the difficulty in establishing the clinical application value and interpreting the results from mNGS. To address this challenge, large-scale clinical investigations are needed to provide insights into the clinical value of mNGS and guide the establishment of guidelines for complete experimental setup. This study retrospectively analyzed plasma mNGS data from 653 subjects, discuss the actual diagnostic effect and clinical application value of the cfDNA method in clinical BSI and other systemic infections, and we propose corresponding diagnosis and treatment suggestions.

## Methods

### Setting and study design

**Figure 1:**
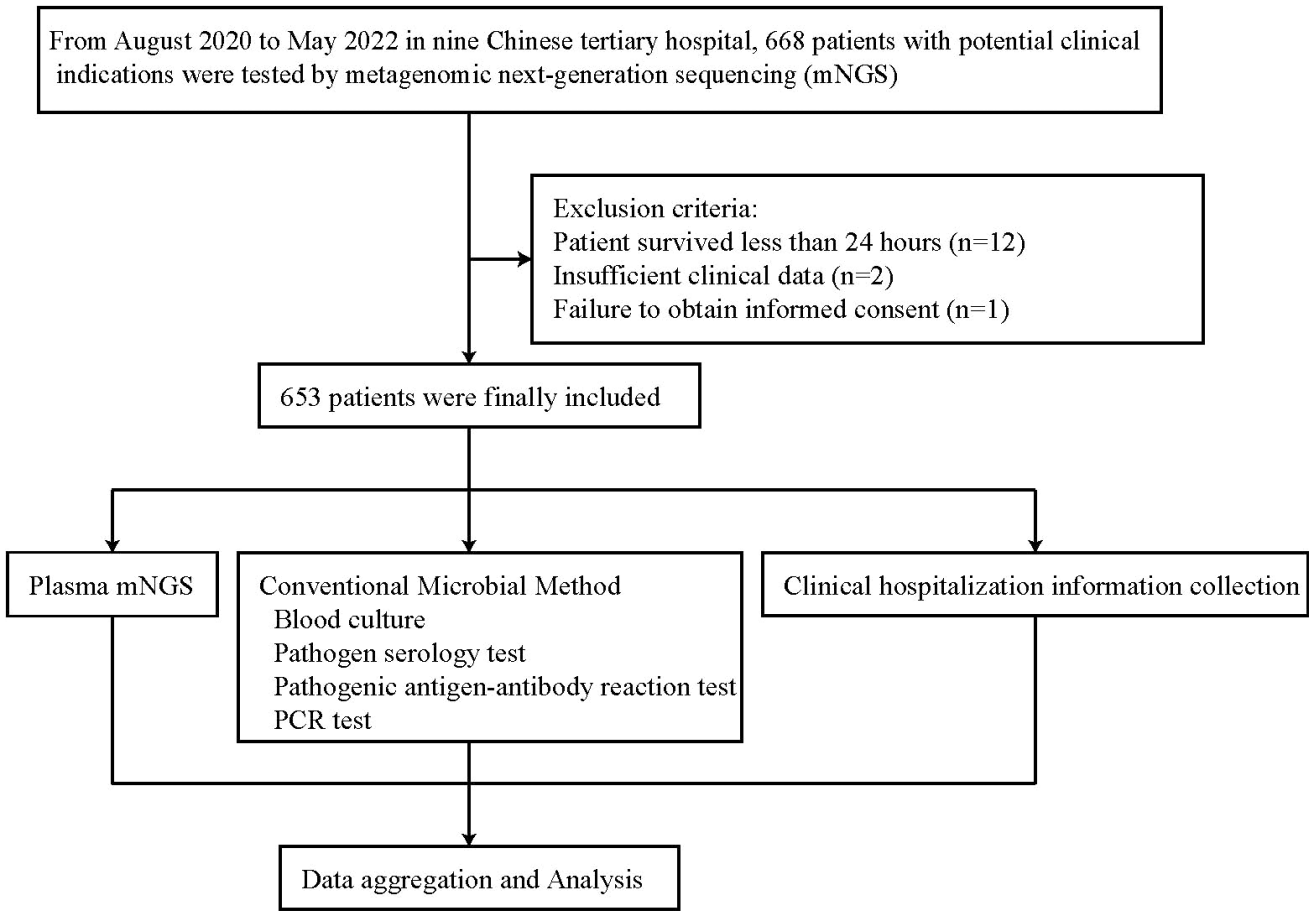
Overview of patient enrollment workflow.

This was a multicenter, retrospective study. We retrospectively studied the medical records of 668 patients who underwent plasma mNGS sequencing in nine medical institutions, including Liaocheng People’s Hospital, between August 2020 and May 2022. The same instrument, library preparation kit, and data analysis pipeline were used at all 9 institutions for plasma cfDNA mNGS. Patients were excluded from the subject group if they died within 24 hours (n = 12), had insufficient clinical data (n = 2), or did not obtain informed consent (n = 1). A total of 653 patients were finally included in the study (**Figure 1**). Plasma mNGS testing and CMTs blood cultures were performed simultaneously (Plasma mNGS and CMT procedure section).

### Plasma mNGS and CMT procedure

The K2EDTA anticoagulant tubes were used for blood specimen collection. All patients underwent plasma mNGS detection, blood culture, and other CMTs methods. Plasma mNGS was performed using the shotgun method on a BGISEQ-50 platform (BGI Genomics, Wuhan, China) with the PMseq^®^ high-throughput detection kit of pathogens (MGI Tech Co., Ltd, Wuhan, China). Prior to its use, the method was validated based on BGI’s general verification process, including running mock specimens to determine limits of detection and specificity. Negative controls were included during library construction and sequencing to control for contamination. Sequence data was based on the BGI self-built microbial genome reference database (see **Supplementary File 1**). Bacterial detection methods generally included microscopic examination, culture, and acid-fast staining for Mycobacterium tuberculosis, among others. Fungal infections were evaluated using 1,3-β-D glucan assay and galactomannan assay, while the Rose Bengal test (RBT) was used for suspected human brucellosis. Common viruses were detected by fluorescence quantitative PCR (qPCR), including Human cytomegalovirus (CMV), Epstein–Barr virus (EBV), Herpes simplex virus (HSV) type 1 and 2, Torque teno virus (TTV), Hepatitis B virus (HBV), JC polyomavirus (JCPyV), BK polyomavirus, and Varicella-zoster virus (VZV). Additional primers were designed and validated by Sanger sequencing in cases where mNGS identified pathogens that were not detected by the above CMT methods.

### Pathogen and clinical data analysis

**Figure 2:**
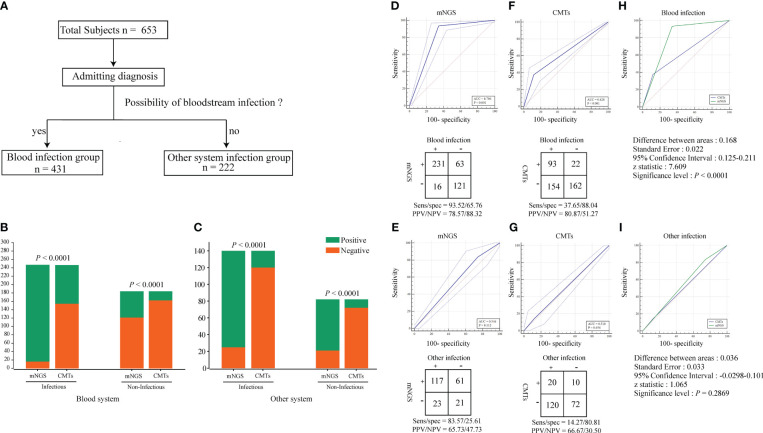
Identification of pathogens by conventional methods and mNGS. **(A)** Group analysis workflow for the samples included in the study. All subjects were divided into two groups based on admitting diagnosis: bloodstream infection group and other systemic infection group. **(B, C)** Positivity rate comparison and concordance analysis between metagenomic next-generation sequencing (mNGS) and CMTs for blood system infection and other system infection samples. The number of positive samples (y-axis) for pairwise mNGS and CMTs testing is plotted against the infectious and Non-infectious (x-axis). The Pearson chi-squared (χ2) test were used for the comparison of categorical data frequencies. P < 0.05 indicates a significant difference. **(D–G)** ROC curves and 2 × 2 contingency tables showing the respective diagnostic performance of mNGS and CMTs for blood system infection and other system infection groups. NPV, negative predictive value; PPV, positive predictive value. **(H, I)** Comparison of the diagnostic efficacy of mNGS and CMTs methods in blood system infection and other system infection groups using ROC curves. P < 0.05 indicates a significant difference.

**Table 1** provides a general reference guide for clinicians considering the use of plasma metagenomic next-generation sequencing (mNGS) in the diagnosis of sepsis and other systemic infections. The table presents potential clinical indications for plasma mNGS, as well as pre-analytical, analytical, and post-analytical considerations that clinicians may find helpful in deciding when to use plasma mNGS testing and how to interpret the results. These recommendations are based on the current literature and expert opinion, and are not specific to the study. To provide additional clarity regarding the criteria used in this study to define “True positive,” “false positive,” and other parameters used for result adjudication, we referred to **Table 2**. This table outlines the theoretical routes for clinical interpretation of plasma mNGS-based detection of sepsis and other systemic infections. The research subjects were divided into two groups based on admission diagnosis: (1) suspected bloodstream infection (BSI) (431 cases); and (2) suspected other systemic infection (222 cases; **Figure 2A**). We separately evaluated the diagnostic efficacy of mNGS and conventional microbiological tests (CMTs) for each group. mNGS detection was considered true positive when the sequencing reads of a pathogenic microorganism detected in plasma were consistent with the clinical diagnosis, including results of microbiological culture and other laboratory tests, as well as the patient’s clinical symptoms and medical history. We used a composite reference method to determine the clinical impact, which included the interpretation of clinical history and all microbiological data, including mNGS findings. To determine the presence of microbial infection, we used comprehensive clinical criteria, including those presented in **Supplementary File 2**. We calculated clinical impact by microorganism and patient, with each microorganism and plasma specimen interpreted as one object, respectively. The calculations considered the diagnostic efficacy of both mNGS and CMTs, as well as the presence or absence of microbial infection, as determined by our comprehensive clinical criteria.

**Table 1:** Analytical considerations for plasma mNGS in diagnosis of sepsis and other system infections.

Potential clinical indications	Preanalytical	Analytical	Postanalytical
• Sepsisbreak • Endocarditis • Severe pneumonia • Invasive fungal infection • Disseminated mycobacterial infection • Local infection with systemic spread • Fever of unkown origin • Other system infections (non-bloodstream) but no optimal specimen • Local and plasma samples sent simultaneously	• 0·3 mL plasma required • Mild/moderate infection not detected by conventional microbiological testing • Severe infection, recurrent infection or mixed pathogen infection are suspected • Suspected infection with atypical or difficult-to-cultivate pathogens • Infections in immunocompromised patients • Select patients with long-term turnaround time that still affects management • mNGS may have advantages over traditional testing turnaround time • Inconsistent or suboptimal antimicrobial treatment response • Tissues or body fluids are preferred for local infections, try plasma mNGS testing when access is not available due to invasive procedures or patient instability • mNGS detection is possible before or after antimicrobial treatment	• Detect cell-free DNA • RNA viruses not detected • Report pathogen sequencing reads, genomic coverage, and relative abundance • No antimicrobial susceptibility or resistance evaluated	• Adapter sequences and low-quality sequences are removed • Set conventional reads threshold: bacteria, viruses, fungi ≥3, parasites ≥100, Mycobacterium tuberculosis, Brucella are set to ≥1 • Interpretation of results by a doctoral-level clinical microbiologist with expertise in mNGS • Organisms frequently occurring interfering background sequences in negative controls are excluded or placed in a separate list of suspected background organisms (informal report content), note that this step may cause mistaken deletion and need to be comprehensively interpreted • Negative mNGS results do not rule out infection • Detection of residual nucleic acid or transient bacteria is usually not judged clinically significant • Distinguish normal human microbiota • Highest abundance organism does not equate to clinical causation • Antibacterial selection consult an infectious disease physician or conduct a multidisciplinary consultation, combining mNGS with other pathogen detection tests

mNGS, metagenomic next-generation sequencing

**Table 2:** Theoretical routes for clinical interpretation from plasma mNGS-based detection of sepsis and other system infections.

Clinical interpretation	Theoretical routes
• True positive	• In sepsis, confirmed positive and primary etiology of disease: e.g., sepsis in patients with blood cultures showing the same bacteria as mNGS testing • In other system infections, the diagnosis is clinically confirmed even without culture validation (no optimal local specimen). e.g., Aspergillus sequences were detected in plasma mNGS, and pulmonary aspergillosis was confirmed by lung CT and G test/GM test
	• Confirmed positive but not the primary cause of disease: e.g., colonization of EBV was detected by mNGS in the plasma of patients with S. aureus-induced sepsis, and EBV was confirmed by PCR
	• Other conditions not confirmed positive but consistent with infectious diagnosis: e.g., in patients with clinically diagnosed disseminated tuberculosis, Mycobacterium tuberculosis was identified on mNGS testing but not by standard microbiological testing
• False positive	• Sequences may originate from interference or contamination: e.g., reagents or environmental contamination during production
	• User normal gastrointestinal/skin/respiratory colonization flora but have no apparent infectious manifestations in patients: e.g., plasma mNGS detected Neisseria. subflava, which is generally considered normal flora of the upper respiratory tract
	Microorganisms with no known clinical significance: e.g., Caballeronia telluris was identified on mNGS in plasma, but its pathogenicity has not been proven
	• In sepsis and other system infections, pathogens identified on plasma mNGS were inconsistent with final clinical diagnosis: Staphylococcus epidermidis was identified on mNGS in plasma without evidence of infection
• True negative	• No positive DNA fragments were detected, and clinical interpretation ruled out sepsis and other system infections
• False negative	• Plasma mNGS did not detect DNA fragments, and sepsis or other system infections were finally diagnosed by other experiments. Local infection pathogen fragments that do not spread into the blood may occur

mNGS, metagenomic next-generation sequencing; CT, computed tomography; G test, β-d-glucan test; GM test, galactomannan test; EBV, Epstein-Barr virus

### Background interference and turnaround time of mNGS

Because mNGS is a very sensitive detection method, contamination may be introduced through the environment, reagents, and processing procedures at any time point during sample collection, nucleic acid extraction, library preparation, and sequencing. To avoid the influence of contamination on the results, we analyzed the main background bacteria and their distribution frequency in mNGS detection. In this study, a total of 70 rounds of mNGS experiments were conducted, with each round including a negative control. In each mNGS experiment, the three background microorganisms (bacteria and fungi were counted once each) with the highest frequency and abundance were counted in both the negative control and negative sample. Subsequently, the dominant background flora were analyzed from 10 rounds of experiments conducted each month and their frequencies were visualized as heat maps. In this study, the turnaround times (TATs) for tests including polymerase chain reaction (PCR), metagenomic next-generation sequencing (mNGS), bacterial culture and identification, and fungal culture and identification were collected and analyzed in the Laboratory Information System (LIS) database. The calculation of TATs began with the receipt of the specimen and ended with the next process or the issuance of the test report.

## Results

### Clinical characteristics of study patients

The study involved 8·3% (54/653) children and 68·8% (449/653) immunocompromised patients. Regardless of clinical relevance, the overall positive detection rate of mNGS was 72·3% (472/653). Compared to patients with a negative mNGS test, patients with a positive mNGS test were more immunocompromised and had a higher rate of severe disease (P < 0·05, **Table 3**). Among the mNGS-positive patients, hematological disease, fever of unknown origin (FUO), and chronic obstructive pulmonary disease (COPD) accounted for the top three identified diseases (**Table 3**).

**Table 3:** Clinical characteristics of study patients (n=653).

Characteristics^1^	mNGS detection^2^		*P* value^3^
	mNGS positve (n=472)	mNGS negative (n=181)	
Age(years), Median (IQR)^4^	57(39·75-69)	53·5(33-66)	0·073
Pediatric patients, n (%)^5^	38(8·1)	16(8·8)	0·734
Gender(male), n (%)	280(59·3)	87(48·1)	0·009
Immunocompromised, n (%)	341(72·2)	108(59·7)	0·002
Intensive care unit, n (%)	100(21·2)	22(12·2)	0·008
Underlying condtion			
Haematological disease, n (%)	134(28·4)	38(21)	0·055
FUO, n (%)	94(19·9)	56(30·9)	0·003
COPD, n (%)	63(13·3)	20(11)	0·430
Solid cancer, n (%)	36(7·6)	6(3·3)	0·044
Cerebrovascular disease, n (%)	33(7)	8(4·4)	0·225
Serious trauma/surgery, n (%)	24(5·1)	8(4·4)	0·725
Immunodeficiency diseases, n (%)	16(3·4)	8(4·4)	0·531
IAI, n (%)	15(3·2)	7(3·9)	0·662
Cirrhosis, n (%)	9(1·9)	8(4·4)	0·071
CHD, n (%)	8(1·7)	5(2·8)	0·382
GI bleeding/perforation, n (%)	7(1·5)	2(1·1)	1
Sepsis, n (%)	6(1·3)	0(0)	0·287
UTI, n (%)	6(1·3)	1(0·6)	0·709
Diabetes, n (%)	5(1·1)	2(1·1)	1
Meningitis, n (%)	4(0·8)	6(3·3)	0·052
CKD, n (%)	4(0·8)	1(0·6)	1
Other, n (%)	8(1·7)	5(2·8)	0·575

mNGS, metagenomic next-generation sequencing; IQR, interquartile range; FUO, fever of unknown origin; COPD, chronic obstructive pulmonary disease; IAI, intra-abdominal infection; CHD, coronary heart disease; GI, gastrointestinal; UTI, urinary tract infection; CKD, chronic kidney disease.

1. For multiple diseases, calculated according to primary disease; 2. mNGS positve is the pathogen fragment reported by sequencing, which may include true positive/false positive for final clinical interpretation. And mNGS positive includes possible bloodstream infection and other system infections; 3. Age P values assess by student t test, and other patient characteristic P values assess by Pearson Chi-Square test or Fisher’s exact test 4. IQR refers to the calculated quartile of 0·25 and 0·75; 5. Pediatric patients refer to age ≤14 years old.

### Comparison between mNGS and CMTs

**Figure 3:**
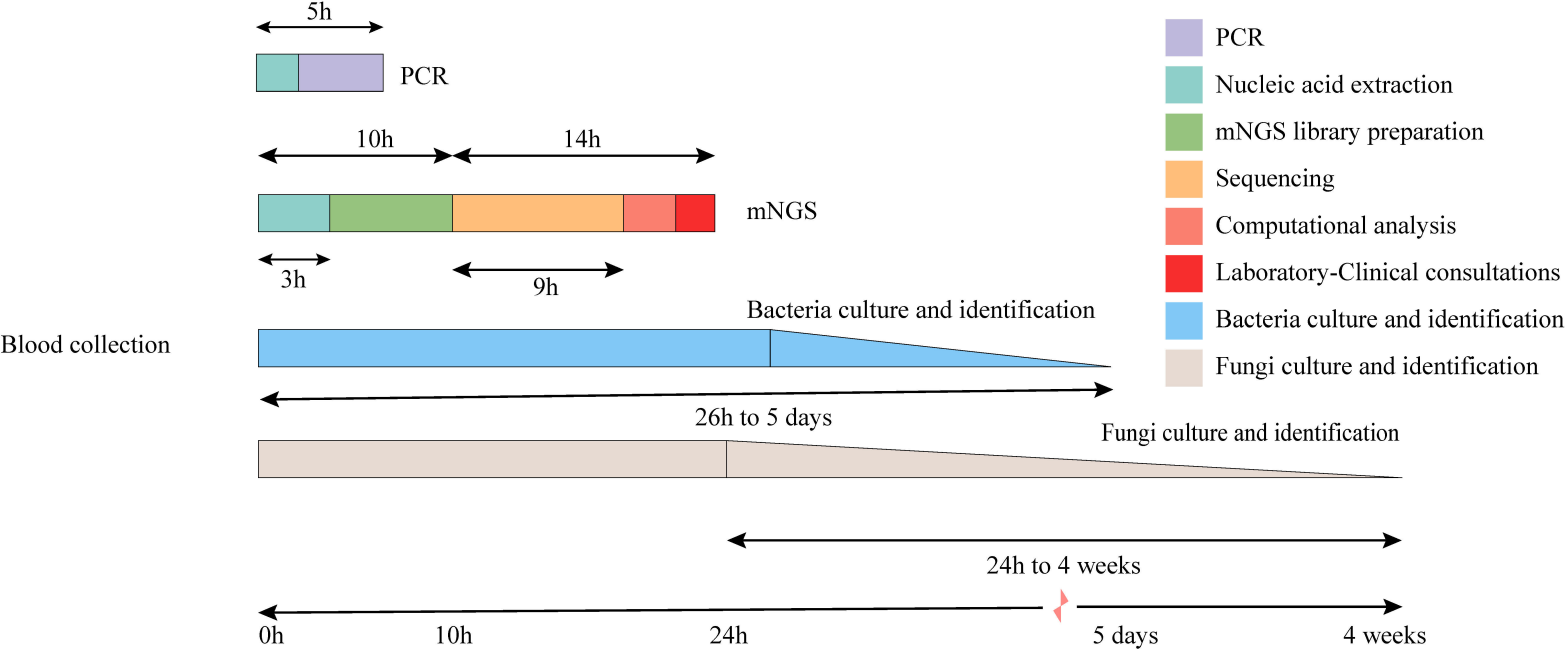
Timeline for mNGS and CMTs. Culture-based pathogen identification can take days to weeks. In comparison, mNGS testing using the BGI sequencing platforms has an overall turnaround time of 24 h.

The TAT analysis showed that mNGS took less time than CMTs, including culture methods. However, mNGS took longer than methods such as PCR and antibody detection (**Figure 3**). In both the BSI and other systemic infection groups (**Figure 2A**), the true positive rate of mNGS diagnosis was greater than that in the CMTs (**Figures 2B, C**). The diagnostic sensitivity of mNGS was also greater than that of CMTs; however, its diagnostic specificity was less than that of CMTs (**Figures 2D–G**). We analyzed the diagnostic performance plasma mNGS and CMTs in these two groups. The results showed that mNGS had a receiver operating characteristic (ROC) curve of 0.796 for suspected BSIs, which was significantly higher than that of CMTs (P < 0·01, **Figures 2D, H**). Both mNGS and CMTs had low diagnostic performance in detecting other systemic infections. The area under curve (AUC) value of mNGS was 0.546 (CMTs = 0.510), with no statistically significant difference between them (**Figure 2I**).

### Pathogen species and infection categories

We detected a total of 735 suspected causative pathogens in the plasma of 653 patients using mNGS, of which 171 plasma samples showed multiple organisms. Among all 735 microorganisms, we detected 380 bacteria (51·7%), followed by viruses (32·0%). The gram-negative bacilli (GNB) were the most common pathogens and directly associated with approximately 50% of sepsis cases. Community-acquired pneumonia, hospital-acquired pneumonia, accounted for the largest proportion of infections among other systemic infections (**Table 4**). Among all unconfirmed infection sequences, viruses accounted for the highest proportion of sequences (69·8%, 164/235), which may be related to the latent of EBV or HSV, although their association with diseases remains unclear. Gram-positive bacteria caused an increase in an unconfirmed infection due to clinical uncertainty in coagulase-negative staphylococci. The detailed distribution of organisms detected in the plasma by mNGS is provided in **Supplementary File 3**. Sequence reads were in the ranges of 3–57969, 3–4454, 3–41266, and 3–914 for bacteria, fungi, viruses, and other rare pathogens, respectively. In addition, we observed a higher number of DNA sequences in clinically relevant cases (**Table 5**). During the study, a total of 26 special pathogens were identified with reads ranging from 3 to 2290 (**Table 6**). Some special pathogens can be confirmed by PCR or culture. However, due to their rarity, certain pathogens cannot be confirmed by other methods, and infection can only be diagnosed by comprehensive clinical interpretation.

**Table 4:** Infection categories pointed to by positive microorganisms in plasma detected by mNGS assay.

mNGS positive (n=735)	Infection categories^1^							
	Sepsis	Other system infections						Unproven infection^2^
		HAP	CAP	IAI	UTI	Meningitis	SSTIs	
Bacteria (n=380)								
GNB(n=237)	139	25	19	6	4	··	··	44
GPB(n=140)	51	10	8	4	··	2	3	62
Mycobacterium(n=3)	2	··	1	··	··	··	··	··
Virus(n=235)	43	12	9	··	··	2	5	164
Fungus(n=107)	22	19	15	5	··	··	··	46
Parasite(n=1)	1	··	··	··	··	··	··	··
Mycoplasma/Chlamydia /Rickettsia(n=12)	3	··	3	··	2	··	··	4

mNGS, metagenomic next-generation sequencing; HAP, hospital-acquired pneumonia; CAP, community-acquired pneumonia; IAI, intra-abdominal infection; UTI, urinary tract infection; SSTIs, skin and soft tissue infections; GNB, Gram-negative bacteria; GPB, Gram-positive bacteria.

1. Multi-site infection is counted by primary infection; 2. Unproven infection consists of two parts. One refers to the true positive results of mNGS verified by other methods, but clinically confirmed not to be the primary cause of disease, such as EB virus detected of mNGS confirmed by PCR. Another part is that only mNGS detected the fragment, but its pathogenicity could not be determined.

**Table 5:** Comparison of distributions of sequencing reads for clinically relevant and irrelevant organisms (lines indicate median values) .

Microorganisms detected by mNGS (n=735)	Reads Range	Median Reads(IQR)	*P* ^1^
Bacteria (n=380)			
Clinically relevant(n=274)	3-57969	98 (7,23)	< 0·01
Clinically irrelevant(n=106)	3-36	5(3,11)	
Virus(n=235)			
Clinically relevant^2^(n=71)	3-41266	69(20,283)	0·03
Clinically irrelevant(n=164)	3-51	8(4,16)	
Fungus(n=107)			
Clinically relevant(n=61)	3-4454	102(32,293)	< 0·01
Clinically irrelevant(n=46)	3-31	6(3,10)	

mNGS, metagenomic next-generation sequencing; IQR, interquartile range; GNB, Gram-negative bacteria; GPB, Gram-positive bacteria.

1. P < 0·05 indicates a significant correlation; 2. Clinically relevant means that the final clinical diagnosis is the causative pathogen, and some viruses (such as EBV) are true positive but the clinical significance of infection is not clear, and it is judged as clinically irrelevant.

**Table 6:** Processing details detected in plasma mNGS for specific pathogens.

Patient No.	Age/Sex	Clinical Comorbidities	Plasma mNGS reasons	Special mNGS species (Reads)	Coexistence of other species (Reads)	Test verification	Diagnose infection	Clinical Impact	Treatment effect	Other detail
**1**	33 y/M	Hepatic insufficiency, enlarged spleen	FUO	Coxiella burnetii(4)	None	Specific antibody(-), PCR(-)	No	Negative	N/A	This pathogen was not considered clinically
**2**	46 y/M	Bronchitis	FUO	Coxiella burnetii(63)	None	Specific antibody(+), PCR(+)	BSI	Positive, New diagnosis and targeted antimicrobial therapy	Yes	After mNGS detected the bacteria, the antibiotics were changed to doxycycline for 7 days, and the symptoms were significantly improved without re-fever. After 7 days of continuous treatment, the effect is as expected.
**3**	42 y/M	COPD, bronchitis	FUO	Coxiella burnetii(25)	None	Specific antibody(+), PCR(+)	BSI	Positive, New diagnosis and targeted antimicrobial therapy	Yes	Patient has a history of work in an abattoir. After mNGS detected the bacteria, the patient was given doxycycline anti-infective treatment for 4 days without re-fever, and continued treatment for 10 days after improvement.
**4**	73 y/F	CKD	FUO	Mycolicibacterium mucogenicum(15)	None	Blood culture(-)	BSI	Positive, New diagnosis and targeted antimicrobial therapy	Yes	According to the clinical manifestations of sepsis, test results were used to replace amikacin in combination with clarithromycin for 4 weeks with expected results.
**5**	64 y/M	Septal perforation repair, MODS, hypertension, pneumonia	FUO	Mycolicibacterium mucogenicum(6)	None	Blood culture(+)	BSI	Positive, New diagnosis and targeted antimicrobial therapy	No	After the mNGS results were reported, the clinical diagnosis was sepsis, and the combination of amikacin and moxifloxacin was applied for 4 weeks. Blood cultures were positive after 3 days.
**6**	49 y/F	Traumatic brain injury, subdural hematoma	FUO	Tropheryma whipplei(2290)	None	PCR(+) and 16S rRNA sequencing(+)	Whipple's disease	Positive, New diagnosis and targeted antimicrobial therapy	Yes	The 16S rRNA sequencing was followed up after mNGS positive, and Whipple's disease was confirmed. Intravenous ceftriaxone was given for 14 days and then discharged to oral trimethoprim/sulfamethoxazole for one year. Vital signs were normal at the 3-month follow-up.
**7**	20 m/F	MODS, CPR, shock	FUO	Clostridium difficile(26)	None	No	No	Negative	N/A	Clinical interpretation considered that the bacteria may be caused by the translocation of intestinal flora.
**8**	47 y/M	Headache, hepatic insufficiency	FUO	Neisseria gonorrhoeae(17)	None	Trace urethral smear(-), culture(+),PCR(+)	UTI	Positive, New diagnosis and targeted antimicrobial therapy	Yes	After mNGS detected the bacteria, PCR and culture of urethral secretions were followed to diagnose infection. The patient was given ceftriaxone + azithromycin for 10 days, and was discharged after improvement.
**9**	69 y/M	Cerebral infarction, hypoalbuminemia, diffuse pulmonary fibrosis	FUO	Legionella pneumophila(3)	Haemophilus parainfluenzae (5)	Trace urinary antigen(+)	CAP	Positive, New diagnosis and targeted antimicrobial therapy	Yes	mNGS test identified pathogen prompted chest imaging and urinary antigen that confirmed infection.Treated with moxifloxacin for 10 days to achieve the effect.
**10**	81 y/M	AML,skin ulceration, hypertension	FUO, Simultaneously mNGS for wound secretions	Nocardia asteroides(3)	None	Blood culture(-),secretion culture(-)	SSTIs	Positive, New diagnosis and targeted antimicrobial therapy	Yes	The pathogen was detected simultaneously by mNGS of blood and pus, and the wound healed after 4 weeks treatment with trimethoprim/sulfamethoxazole + imipenem.
**11**	88 y/F	COPD,gastritis	Intolerance to bronchoalveolar lavage	Mycobacterium tuberculosis(3)	None	IGRA(+)	CAP	Positive, New diagnosis and targeted antimicrobial therapy	No	Xpert MTB/RIF was not performed due to intolerance to bronchoalveolar lavage. Combining chest imaging and IGRA(+), antibiotics have been changed, but the patient's underlying condition is ineffective and died.
**12**	55 y/F	HIV/AIDS, hepatitis B cirrhosis, severe anemia	Suspected special pathogen infection	Leishmania donovani(914)	None	Bone marrow smear(+)	BSI	Positive, New diagnosis and targeted antimicrobial therapy	Yes	Lived in forest area for 10 years, HIV/AIDS for 4 years, and the pathogen was treated with amphotericin B liposome according to the recommended protocol (curr opin infect dis 26:1, 2013).
**13**	57 y/F	SS, pneumonia, cholecystitis and gallstones	FUO	Rickettsia felis(57)	None	PCR(-)	No	Negative	N/A	Clinical interpretation of fever considered primary immune system disease.
**14**	39 y/F	Moderate anemia	FUO	Rickettsia felis(493)	None	PCR(+)	BSI	Positive, New diagnosis and targeted antimicrobial therapy	Yes	Patient fed cat pet at home before admission. Doxycycline was used for 7 days after mNGS detected the bacteria, and symptoms were significantly improved.
**15**	59 y/M	CML, myelofibrosis, myeloproliferative tumor hypertension	FUO	Rickettsia felis(403)	None	PCR(-)	BSI	Positive, New diagnosis and targeted antimicrobial therapy	Yes	The patient had been scratched by a stray cat 10 days earlier. Treated with doxycycline for a week and discharged.
**16**	62 y/F	Ovarian cancer	FUO	Mycoplasma hominis(21)	Staphylococcus hominis (21)	Trace urethral PCR(+)	UTI	Positive, New diagnosis and targeted antimicrobial therapy	Yes	Follow up the patient's urinary tract PCR test to confirm the diagnosis, symptoms disappeared within 7 days of azithromycin.
**17**	59 y/M	Cerebral infarction, pneumonia, hypertension	FUO	Mycoplasma hominis(9)	Corynebacterium striatum (2659), Acinetobacter baumannii (160), Klebsiella pneumoniae (126), Enterococcus faecium (90), Candida albicans (201)	No	No	Negative	N/A	Clinical interpretation was an accompanying fragment, with no clinical significance for infection.
**18**	45 y/F	Lung cancer with multiple metastases	FUO	Mycoplasma pneumoniae(3)	None	Serum IgG(+)	CAP	Positive, New diagnosis and targeted antimicrobial therapy	Yes	Experience gave ciprofloxacin anti-infection, and changed azithromycin therapy after the detection of Mycoplasma pneumoniae by mNGS. Then patient recovered after 7 days.
**19**	63 y/M	CML	FUO	Mycoplasma pneumoniae(6)	Burkholderia cepacia (12619), HSV1-1 (11)	Serum IgG(+)	CAP	Positive, New diagnosis and targeted antimicrobial therapy	Yes	Blood culture detected B. cepacia and was treated with ciprofloxacin. The patient's body temperature rose repeatedly during treatment. Combined with mNGS, chest imaging and M. pneumoniae antibody detection followed by azithromycin treatment, recovery after 4 days
**20**	62 y/M	RA, CKD, intestinal obstruction, shock, hypertension	FUO	Ureaplasma urealyticum(21)	None	No	No	Negative	N/A	Comprehensive clinical interpretation did not consider the organism to be pathogenic
**21**	31 y/F	Puerperal infection,pneumonia, pleural effusion, intestinal obstruction	FUO	Ureaplasma parvum(6)	None	Trace urethral PCR(+)	UTI	Positive, New diagnosis and targeted antimicrobial therapy	Yes	Ceftriaxone was empirically selected according to Enterobacteriaceae, and then upgraded to meropenem, which was ineffective. The patient had intermittent fever, the same pathogen was confirmed by plasma mNGS and urethral PCR. With azithromycin for 3 weeks, urethral symptoms disappeared.
**22**	53 y/F	AML	FUO	Rickettsia prowazekii(4)	Candida albicans (29), EBV (6)	PCR(+)	BSI	Positive, New diagnosis and targeted antimicrobial therapy	Yes	It was found that the patient had wild jungle travel experience. After comprehensive analysis of the examination results, the symptoms disappeared after 10 days of treatment with doxycycline.
**23**	57 y/M	Gastrointestinal perforation, hypothyroidism, iatrogenic Cushing, steroid-induced diabetes	FUO	Mycoplasma orale(3)	Corynebacterium striatum(12), HBV (335), CMV (4)	No	No	Negative	N/A	The clinical interpretation suggested that the bacteria may be caused by translocation of the normal flora.
**24**	71 y/F	COPD, acute pyelonephritis, hypertension	Intolerance to bronchoalveolar lavage	Chlamydia psittaci(3)	None	No	CAP	Positive, New diagnosis and targeted antimicrobial therapy	Yes	Empirical antimicrobial therapy was ineffective. The patient was asked about life history of raising birds. Combined with mNGS and chest CT atypical pneumonia, the body temperature was normal after 4 days of azithromycin.
**25**	66 y /M	Hypertension	Local tissue intolerance to puncture	Brucella(6)	EBV(13)	Blood culture(-), RBPT(+)	BSI	Positive, New diagnosis and targeted antimicrobial therapy	Yes	Sacroiliac joint pain, suspected infection on imaging. mNGS results prompted RBPT(+), followed by doxycycline + rifampicin for 3 months, plus gentamicin for the first week.
**26**	33 y/M	Hepatitis B cirrhosis, hypersplenism	FUO	Brucella(9)	None	Blood culture(-), RBPT(+)	BSI	Positive, New diagnosis and targeted antimicrobial therapy	Yes	The patient has a history of raising sheep at home. Combined with mNGS and RBPT(+), he was given doxycycline for 6 weeks and gentamicin for the first week.

mNGS, metagenomic next-generation sequencing; M, male; F, female; y, years; m, month; COPD, chronic obstructive pulmonary disease; CKD, chronic kidney disease; MODS, multiple organ dysfunction syndrome; CPR, cardiopulmonary resuscitation; AML, acute myeloid leukemia; HIV, human immunodeficiency virus; AIDS, acquired immunodeficiency syndrome; SS, sjogren syndrome; CML, chronic myeloid leukemia; RA, rheumatoid arthritis; FUO, fever of unknown origin; EBV, Epstein-Barr virus; HBV, hepatitis B virus; CMV, human cytomegalovirus; HSV, herpes simplex virus type; RBPT, rose bengal plate agglutination test; BSI, bloodstream infection; UTI, urinary tract infection; CAP, community-acquired pneumonia; IGRA, interferon-γ release assay; N/A: not applicable.

### Clinical impact and relevance of mNGS

Of all 472 mNGS-positive patients, clinical management of 203 (43·0%) patients was positively affected by mNGS (e.g., new pathogens identified and antibiotic regimen changed) (**Table 7**). A total of 269 (57·0%) patients did not change their treatment regimen (e.g., pathogen detection of unknown significance, mNGS results were slower than CMT results, or previous antibiotic regimen covered the pathogen) (**Table 7**). Furthermore, 92 patients received a modified clinical management regimen, including de-escalation of antibiotics and avoidance of invasive tests such as tissue biopsy, based on negative mNGS test results suggesting a low likelihood of pathogen infection. Of the 653 patients who underwent plasma mNGS testing, 295 (45·2%) benefited from the technology. Among the 735 microorganisms detected by mNGS, 489 (66·8%) were not detected by CMTs. Of these, 232 (47·4%) microorganisms led to a change in the clinical management of patients that ultimately benefited the patients.

**Table 7:** Clinical impact of mNGS in plasma by patient (n=653) and by microorganism (n=735).

mNGS result	Change in management	Clinical impact^1^	By patient^2^ (n=653), count(%)
Positive	Yes	New diagnosis based on mNGS result and not conformed by CMTs, mNGS result enabled initiation of targeted antimicrobial therapy^3^	165(25·3)
		Earlier diagnosis based on mNGS result, later confrmed by CMTs, mNGS result enabled initiation of targeted antimicrobial therapy	38(5·8)
Positive	No^4^	mNGS result showed new organism, but antibiotics and clinical plan were not changed	162(24·8)
		mNGS result confrmed CMTs diagnosis and no additional action	107(16·4)
Negative/false positive^5^	Yes	No mNGS result confrmed clinical diagnosis, de-escalated treatment of antimicrobial	65(10)
		No mNGS result confrmed clinical diagnosis, avoid invasive surgical biopsy	27(4·1)
Negative/false positive	No	mNGS test result was negative, no action taken	89(13·6)
mNGS result	Change in management	Clinical impact^1^	By microorganism^2^ (n=735), count(%)
Positive	Yes	New diagnosis based on mNGS result and not conformed by CMTs, mNGS result enabled initiation of targeted antimicrobial therapy^3^	232(31·6)
		Earlier diagnosis based on mNGS result, later confrmed by CMTs, mNGS result enabled initiation of targeted antimicrobial therapy	52(7·1)
Positive	No^4^	mNGS result showed new organism, but antibiotics and clinical plan were not changed	257(35)
		mNGS result confrmed CMTs diagnosis and no additional action	194(26·4)

mNGS, metagenomic next-generation sequencing; CMTs, conventional microbiological tests.

1. Clinical impact were defined by a composite reference method, interpreting clinical history and all microbiological data, including mNGS findings; 2. Clinical impact were calculated by microorganism (each microorganism is interpreted as a whole) and by patient (plasma specimens from each patient are interpreted as a whole); 3. In the case of this study, since the implementation of mNGS with other system infections were often due to the inability to obtain appropriate local specimens for testing, the emerging pathogens were considered new discoveries. This was more likely to point to a new infection diagnosis, increasing the counts for this item (including other system infections 93 by microorganism and 67 by patient); 4. Non-replacement due to antimicrobial coverage of the current pathogen was included in this item; 5. If both de-escalated treatment of antimicrobial and avoid invasive surgical biopsy are present, only the primary one will be recorded.

### Background microorganisms

**Figure 4:**
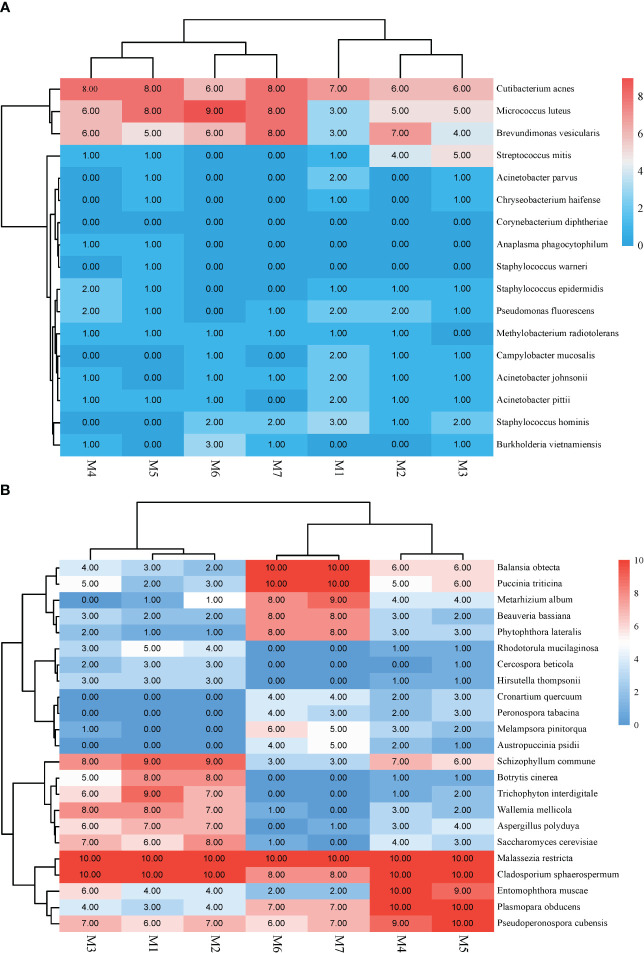
Heat map illustrating the common background Microorganisms in mNGS. **(A)** Background bacteria. **(B)** Background fungi.

In this study, bacteria mainly caused background interference and reads ranged between 10 and 117. The top three bacteria were Cutibacterium acnes, Micrococcus luteus, and Brevundimonas vesicularis (**Figure 4**).

## Discussion

Currently, mNGS is not recommended for routine use in mild/moderate infections due to its high cost (Chiu and Miller, 2019). However, it is necessary to carry out adequate development in complex and critically ill patients (Simner et al., 2018). The external clinical manifestations of FUO and other various diseases (Goggin et al., 2020; Yan et al., 2021), especially for different immunocompromised conditions (Carbo et al., 2022), are often detected by plasma mNGS. In our investigation, the utilization of plasma mNGS was found to be effective in identifying instances of bloodstream infections, particularly those caused by uncommon pathogens, thus highlighting the utility of this technology. In addition, our study provided reasonable inclusion criteria that can effectively guide clinicians in selecting tests or assays. The findings also confirmed that immunocompromised and ICU patients were more likely to obtain positive mNGS results. Similar studies have shown that plasma mNGS of ICU samples are effective in diagnosing bacterial BSI (Yan et al., 2021). Additionally, we note that mNGS has advantages over CMTs, especially for fungi and mycobacteria, which further expands the clinical indications of this technology. Hogan et al (Hogan et al., 2021) reported that plasma mNGS provided results faster for three fungal diagnoses, leading to earlier optimization of antifungal therapy. Because our mNGS protocol requires ~ 24 hours of TAT, patients with longer hospital stays may benefit from mNGS, potentially impacting their clinical management plan. Furthermore, we believe that when the clinical treatment response is inconsistent with CMTs, it is often a misjudgment of the infected pathogen, and the timely implementation of mNGS will improve diagnostic efficiency.

Another potential application of plasma mNGS is the identification of other non-blood systemic infections, which is a feasible and new attempt. Li et al (Li et al., 2022) reported that among 109 patients with an abdominal infection, 61 positive cases were detected and clinically confirmed by plasma mNGS. In other reports, plasma mNGS has been helpful in the diagnosis of patients with endocarditis (Kondo et al., 2019; Hogan et al., 2021). In addition, pneumonitis and invasive mycosis have also progressed experimentally by using plasma mNGS (Hong et al., 2018; Farnaes et al., 2019). However, the significance of guiding clinical infection management is still controversial due to the small number of cases using this technology.

A novel and comprehensive study on the application of plasma mNGS method for the detection of systemic infections was conducted. This study differs and surpasses some of the previous studies published in this field in several aspects. Blauwkamp et al. reported a blood cfDNA sequencing-based diagnostic method (Blauwkamp et al., 2019), but their pathogen database was limited to bacteria and fungi, while a larger and more comprehensive database encompassing more types and numbers of microorganisms, such as viruses, parasites, mycoplasma, chlamydia, etc., was used in this study. Their method was only tested on simulated samples, while the method in this study was validated on clinical samples, and compared with conventional methods, demonstrating high consistency and accuracy.

In the realm of infectious disease diagnostics, metagenomic next-generation sequencing (mNGS) stands as a beacon of innovation, offering a broad-spectrum pathogen detection capability that transcends the limitations of traditional culture-based methods (Loman and Pallen, 2015). The principal advantage of mNGS lies in its unparalleled sensitivity and specificity, enabling the identification of a diverse array of pathogens, including bacteria, viruses, fungi, and parasites, from a single sample (Chiu and Miller, 2019). This holistic approach is particularly beneficial in cases of polymicrobial infections, where multiple pathogens coexist, often eluding conventional diagnostic techniques. Moreover, mNGS is adept at detecting novel or rare pathogens, thereby expanding the diagnostic horizon significantly. However, this technology is not without its limitations. The interpretation of mNGS data can be challenging, given the complexity of distinguishing pathogenic sequences from background microbial DNA, particularly in cases of low-level or transient bacteremia (Meyer et al., 2022). Additionally, the high cost and technical expertise required for mNGS limit its widespread adoption in routine clinical practice. Furthermore, while mNGS offers rapid turnaround times compared to traditional cultures, the need for specialized equipment and bioinformatics support can introduce delays (Hasman et al., 2014). Thus, while mNGS heralds a new era in infectious disease diagnostics, its integration into clinical practice necessitates a balanced consideration of its strengths and limitations, underscoring the need for continued research and development in this dynamic field (Rhoads et al., 2012; Lecuit and Eloit, 2015).

In this study, our sizeable clinical database diagnosed 34 cases of fungal pneumonia and one case of tuberculous pneumonia by plasma mNGS, which provides another option for examination in patients who cannot tolerate bronchoalveolar lavage. In addition, we found that the nucleic acid fragments of urinary pathogens by nucleic acid fragments in plasma, such as *Neisseria gonorrhoeae*. Nevertheless, as demonstrated in this study, the diagnostic accuracy of plasma mNGS for infections in other body systems is relatively low. Tissue or body fluids are still recommended for local infections due to the high local concentration of pathogens. Therefore, deriving local infections from plasma mNGS can only be used as an alternative since not all pathogenic nucleic acids can spread to the blood.

The mNGS technique exhibits high sensitivity compared to CMTs, which was confirmed in our study. A similar study examined the value of mNGS in predicting BSI in childhood leukemia patients and demonstrated that the assay had a predictive sensitivity and specificity of 75% and 82%, respectively (Goggin et al., 2020). We believe this is also related to patient inclusion criteria, as mNGS procedures are often performed only when there is a high suspicion of infection in the current situation. The decreased specificity of mNGS compared to conventional molecular techniques can result in incorrect diagnoses and treatments in clinical settings. To address this, mNGS results are validated using other techniques, and improvements in mNGS technologies and bioinformatics algorithms are ongoing to improve specificity. However, it is still important to note that “true detection” does not necessarily mean “true infection,” Pathogens (such as EBV) confirmed by PCR in this study cannot be used to guide clinical management directly due to their unclear infection characteristics. A report on the use of mNGS in 386 patients with community-acquired sepsis of unknown etiology in Vietnam demonstrated that a human-infectious virus was detected in 32/384 serum samples. Whether there is a direct clinical association with sepsis remains unclear (Anh et al., 2019). In addition, opportunistic pathogens with unconfirmed infection, such as coagulase-negative staphylococci, have not been shown to have an effective clinical impact. In addition, the presence of multiple mixed pathogens in our study plasma specimens and the relationship between them is very complex. Zanella et al (Zanella et al., 2021) performed mNGS analysis on 25 adult allogeneic hematopoietic stem cell transplantation recipients and detected sequences from ≥ 3 different viruses in 16/25 patients. The relationship between pathogens of different species remains to be determined. The read abundance of different pathogens can be used to interpret disease species load (Carbo et al., 2022), but only for homogeneous pathogens. Different pathogens display distinctive cell wall characteristics and this impacts the efficiency of nucleic acid extraction; thus, a high abundance of organisms does not necessarily equal clinical causation. Therefore, when clinicians report multiple pathogens from a single mNGS specimen, clinical decision-making needs to comprehensively interpret the report based on microorganisms’ nucleic acid acquisition efficiency and pathogenicity.

Views on the impact of mNGS on the final clinical protocol vary and are related to differences between studies, including patient populations, study designs, and definitions used to determine clinical impact. Data from the Children’s Hospital of Chicago showed that mNGS was considered to provide clinically relevant information in 56% of submitted samples (Rossoff et al., 2019). Wilke et al (Wilke et al., 2021) conducted a retrospective chart review of 142 cell-free mNGS test results from a tertiary pediatric hospital and confirmed that 32·4% of the results were directly applicable to clinical management change. In contrast, Niles et al (Niles et al., 2020) showed that in most cases, little or no additional value was gained when mNGS was ordered concurrently with routine testing. A good clinical correlation rate was obtained in our clinical practice due to the following aspects.

First, many patients underwent mNGS testing even when CMT results were negative, which was found to be more likely to result in a positive diagnosis. Second, some subjects in this study with local infections were not able to provide local specimens due to limitations and culture testing was not performed. Reliance on plasma mNGS was more effective in detecting new infections. Third, the value of mNGS was demonstrated in the discovery of pathogenic bacteria in follow-up tracing, such as the case of urinary tract infection caused by *Ureaplasma parvum* that we observed. Fourth, the importance of mNGS in clinical practice is reflected in the timely diagnosis of patients, such as eliminating the possibility of co-infections in rheumatism patients. These factors validate the clinical impact of mNGS, primarily through the effective application of antibiotics. Because the cause of infection in patients was not identified in the early stage, and increasing antibiotics were used, the misuse of antibiotics was effectively avoided after mNGS detected the pathogen. Similar studies have confirmed that the positive effects of mNGS are mainly driven by the de-escalation of antimicrobial therapy (Farnaes et al., 2019). In addition, we found that change in clinical management rates were lower than the actual mNGS proven infection rates, which is likely related to the fact that many patients have empirically selected antimicrobials that already cover current pathogens.

This study encountered limitations in the application of the mNGS technique. Firstly, variations in mNGS platforms could result in disparities in the number of reads and levels of abundance for the same specimen, leading to disparate interpretations and incongruent final outcomes. Even when utilizing the same platform, modifications to the amount of sequencing data per sample can impact the reads obtained from the specimen (Diao et al., 2022). Our findings were in line with prior studies, which indicated that high read counts are indicative of infection and that increasing the cutoff value might hypothetically elevate the clinical compliance rate. However, it should be noted that such an increase could also reduce analytical sensitivity and potentially eliminate some true positive results (Carbo et al., 2022). To ensure adequate sensitivity of detection, a standard control sample was sequenced to a depth of 20 million reads on our platform. The presence of infection was also established with just three reads, demonstrating that there is not a single universally accepted threshold. On the other hand, it is possible that a pathogen may go undetected if its sequencing reads are very low (less than three), which reflects its low concentration in plasma. In cases where other sources of disseminated infection are reflected in plasma mNGS, this low yield of sequencing data becomes less effective. Such low pathogen concentrations are also unlikely to be confirmed by culturing methods. While augmenting the sequencing depth per sample might increase the detection rate, it is also crucial to consider the increased cost and potential increase in background noise.

Second, the organisms detected by mNGS experiments may originate from preanalytical processes or from clinically unrelated organisms (Lee et al., 2020). Although these conditions are generally considered false positives, it is sometimes difficult to distinguish between true infection or colonization by normal flora. Particularly in immunocompromised patients, the human microbiota is more likely to translocate into the bloodstream leading to infection (Horiba et al., 2021). Studies have demonstrated that contaminating nucleic acids can be quantified to identify false-positive fragments using the mNGS process (Zinter et al., 2019; Jing et al., 2021), but different sequencing platforms and reagents result in different contaminating sequences (Diao et al., 2022). In this study, we established our own background microbial library, which was beneficial to reduce the interference of common contaminating sequences. It should be noted that different laboratories need to establish their own background bacterial libraries and update them regularly, especially when there are changes in instruments or reagents.

Third, despite the excellent true-negative parameters provided by the mNGS test (Lee et al., 2020), there were still some false-negatives in our experiments, especially for plasma viruses that were lower compared with the real-time PCR method. In addition, this study did not involve the detection of RNA, which may miss some RNA viruses in patients with unexplained fever. Software processing of sequencing data can also cause false negative results. Because the exported sequencing data are often sorted according to reads or abundance, high-interference sequences can cause the true pathogenic bacteria to rank lower and not be automatically exported. Data processing errors can also occur, such as database alignment errors. Positive results were deleted by mistake due to the same organism deriving from the negative control. At present, it is still difficult to formulate specific screening rules for each sample. When the data, automatically exported by the software, do not match the clinical data, the logout list of each sequencing run should be carefully reviewed. Microbiologists can identify sequences that may be clinically significant. Additionally, mNGS testing in our subjects could be performed before and after antimicrobial treatment. In theory, dead organisms have little effect on molecular detection methods, but the reduction in the overall DNA amount after the number of pathogens is reduced may also have an impact on the results. Fourth, although some clinical effects indicate positive manifestations, it does not rule out that in certain circumstances, physicians have no choice but to “believe” the mNGS results, which may not necessarily be accurate, and make changes in clinical antimicrobial therapy accordingly. Finally, we believe that there is still a large oversight in assessing antimicrobial susceptibility or resistance using current methods and warrants future investigations.

## Conclusion

The mNGS test is technically complex and no guidelines are available to help with the interpretation of the results. Before the technology is further developed and running costs reduced, it is necessary to emphasize the establishment of strict access standards, control of various parameters of the detection process (including wet and dry experiments), and professional review and multidisciplinary discussion during interpretation. This will ensure improved pre-screening probabilities and yield clinically meaningful, value-added data.

## Data availability statement

The data presented in the study are deposited in the CNGB Sequence Archive (CNSA) of China National GeneBank DataBase (CNGBdb), accession number CNP0003204 (https://db.cngb.org/search/project/CNP0003204/).

## Ethics statement

The studies involving humans were approved by Medical Ethics Committee of Liaocheng People’s Hospital. The studies were conducted in accordance with the local legislation and institutional requirements. The participants provided their written informed consent to participate in this study.

## Author contributions

FP: Conceptualization, Writing – original draft, Writing – review & editing. WX: Data curation, Writing – original draft, Writing – review & editing. HZ: Data curation, Investigation, Writing – original draft. SC: Data curation, Writing – original draft. YT: Data curation, Writing – original draft. JF: Methodology, Writing – original draft. ZY: Data curation, Writing – review & editing. PS: Formal Analysis, Writing – original draft. QX: Writing – review & editing. QZ: Writing – review & editing. XJ: Writing – review & editing. CW: Data curation, Formal Analysis, Investigation, Methodology, Software, Writing – original draft, Writing – review & editing.
