# Supplementary material for: Comprehensive evaluation of plasma microbial cell-free DNA sequencing for predicting bloodstream and local infections in clinical practice: a multicenter retrospective study
Source: Front Cell Infect Microbiol. 2024 Jan 4;13:1256099. doi: 10.3389/fcimb.2023.1256099 (PMC10868388; doi:10.3389/fcimb.2023.1256099)

**Supplementary file**

**Supplementary file 1**

**File format:.doc**

**Title of data: Supplementary Methods**

**Description of data: The whole process of mNGS.**

**Supplementary file 2**

**File format:.doc**

**Title of data: Supplementary Tables**

**Description of data:Comprehensive Clinical Criteria for Determining Microbial Infection**

**Supplementary file 3**

**File format: .xls**

**Title of data: Distribution of organisms in plasma detected by mNGS**

**Description of data: All the original sequencing data correspondences to infection types, including Gram-positive bacteria, Gram-negative bacteria, fungi, Mycobacteria, rare pathogens, etc.**

**Supplementary file 1**

**Supplementary Methods**

**Sample Preparation**

The whole blood samples were placed in a pre-cooled refrigerated centrifuge at 1600G for 10 min. The 300μl supernatant (plasma) of the prepared sample was subjected to DNA extraction.

**Quality Control**

A negative control consist of Hela cell and a positive control consist of *Acinetobacter baumannii* ATCC 19606 (10^5^ CFU/ml of bacteria suspended in Ringer's solution) were analyzed with each batch. And specific synthetic sequence was added into each specimen as internal standard to monitor the whole process.

**DNA Extraction and Purification**

Cell-free DNA was extracted using the column extraction kit TIANamp Micro DNA Kit (DP316, TIANGEN Biotech, Jiangsu, China). This process does not introduce a microbial DNA enrichment step. The concentration of the extracted nucleic acid was determined by the Qubit dsDNA HS Assay kit using Qubit Fluorometer 4.0 (Invitrogen, Singapore).

**Next-Generation Sequencing**

DNA libraries were prepared using the DNA construction kit (MGI Tech Co., Ltd, Wuhan, China) after end-repair, adapter-ligation, and PCR amplification. Library construction and pooling were performed in strict accordance with the instructions. The input amount of nucleic acid for single library construction is 1-100 ng, and the number of PCR cycles is 16. Use nucleic acid-free water as the library eluate. The constructed library was quantified by Qubit 4.0 using ExKubit dsDNA Assay Kit (Invitrogen) and Agilent 2100 (Agilent Technologies, Santa Clara, CA) for fragments length control. The library concentration quality control standard was double-stranded DNA concentration >1.0 ng/μl, fragment size ~280 bp. Then convert to a single-stranded circular DNA library by DNA denaturation and circularization. Qualified library was pooled and prepared DNA nanoballs (DNB) with a rolling circle amplification (RCA) manner which was unique to BGI Genomics.

The concentration of DNB was determined with the Invitrogen Qubit ssDNA Assay Kit using Qubit 4.0, and the range of 8-40ng/μL is qualified. According to the combined probe-anchored polymerization sequencing method, the PMseq infection pathogen high-throughput detection kit (MGI Tech Co., Ltd, Wuhan, China) was used to load DNB into the sequencing reaction universal kit (MGI Tech Co., Ltd, Wuhan, China), and sequenced on the BGISEQ-50 platform (BGI Genomics, Wuhan, China). The reads sequencing strategy is single-end, the read length is 50 (nt), the index sequencing strategy is double-ending, and the index read length is 10 (nt).

**Metagenomic Data Bioinformatics Analysis**

First, the raw data of sequencing is processed, and high-quality data is obtained after removing adapter sequences and low-quality sequences. Then the high-quality data from sequencing were mapped to the human genome (hg19) using Burrows-Wheeler Alignment (BWA)[1], annotated and striped human genome data. The remaining sequencing data were simultaneously aligned to four self-built microbial genome databases, including viruses, bacteria, fungi, and parasites, by BWA to generate the original mapping list. The self-built microbial genome reference database currently contains 6,039 bacteria, 2,700 DNA viruses, 1,064 fungi, 234 parasites, and 137 mycoplasma/chlamydia, all associated with human disease. Reference genomes in the database were downloaded from the National Center for Biotechnology Information (ftp://ftp.ncbi.nlm.nih.gov/genomes/). Count and normalize the number of uniquely aligned reads to obtain stringently mapped read numbers to species and stringently mapped read numbers to genus. CovRate and Depth were calculated for each microorganism using BEDTools[2]. The relative abundance of species, the relative abundance of genus, the absolute abundance of species, and the absolute abundance of genus were calculated according to the algorithm of BGI software. The software parameter flow of data preprocessing and database comparison, see Supplementary Method Table SM1 and Supplementary Method Table SM2. The total number of mNGS reads in all samples was normalized before data output and report interpretation.

**Data Output and Report Interpretation (See** **Supplementary Method Figure SM1)**

- **Data Volume Standard**

After the bioinformatics analysis is completed, check the data volume (Total) and the human-derived ratio (Hg19_rate) of each sample. Generally, the human-derived ratio is required to be more than 90%. The single-sample data yield standard is 15M-25M reads to grantee the accuracy of the ultimate result. If >25M, intercept the upper limit data volume. If 10-15M, samples with sequencing depth=1 can be interpreted normally. For samples with a sequencing depth >1, an interpretation report can also be issued with a clear positive result. For samples with sequencing depth >1 and no clear positive results, the experimental data needs to be reviewed. If it is determined that the data volume is low due to the sample itself, the report should be interpreted according to the normal interpretation process in principle. Other reasons (experimental input, construction library concentration, etc.) need to be remeasured at the corresponding step. If the amount of data <10M, the blood should be re-tested once. After retesting, if the two analysis results are consistent and there is no suspected pathogenic bacteria, a negative result will be given. After retesting, if the two analysis results are consistent and there are suspected pathogenic bacteria, try to interpret and add the suspected pathogenic bacteria to the list.

- **Detection Sequence Reads Threshold**

The automatic threshold screening rules of the software are as follows, bacteria/virus/fungus/rare pathogen species level reads ≥3 reads, mycobacterium tuberculosis, brucella genuslevel reads ≥1 reads, and parasite species level reads ≥100 reads. The bacteria in the report list show top 20, and the rest of the columns are top 10. The sorting rule in the result list is that the bacteria are sorted at the genus level first and then at the species level. Viruses/fungi/parasites are sorted in descending order of species level. Note that when checking the detected list, especially the bacteria list, the genus ranking may not be completely correct, and the species ranking of bacteria needs to be checked. The descending ranking of covrate is mainly used, and the descending ranking of reads is supplemented. When it is difficult to determine, the coverage, that is, the number of base pairs in the detected sequence, shall prevail (the numerator is the number of detected base pairs, and the denominator is the total number of bases in the genome of the pathogen in the database). In special cases, the pathogen is clinically highly suspicious, and the whole or most of the microorganism lists have low values (detection reads 1 or 2, parasite < 100). Comprehensively analyze the detection situation of the sample, check the same run, negative control, recent samples without the same pathogen, exclude strong positive interference, and then consider adding.

- **Suspected Interfering Background Microorganisms**

Two methods were used to confirm whether the detected microorganisms were background microorganisms. The first is to check the detection of the microorganism in the negative control samples, samples of the same type, and other samples in the same Run. The other is to check the detection of the microorganism in similar samples within a week. Note that changes in the reagents used in the experiment and changes in climate will cause changes in the background bacteria. And the negative control sample needs to be free of common pathogenic bacteria and no bacteria with a particularly high content. Once the organisms are determined to be common laboratory background bacteria will be deleted. Microorganisms that cannot be identified can be added to the background bacteria list as appropriate. In addition, we record suspected background microorganisms and regularly update the background library.

- **Detection Parameters of Pathogenic Microorganisms**

Ideally, the number of reads for the causative pathogen is above the threshold and exceeds the detection value of the negative control sample. The detection results of species with high Reads, low CovRate and high Depth are not credible. In addition, when there are strong positive detections in the same run, other samples are detected with different degrees of contamination and "false positives" need to be deleted from the detection list. When it is difficult to distinguish strong positive contamination from the detection of the sample itself, resequencing or reconstruction of the library can be used to verify the results.

**Supplementary Methods References**

1. Li H, Durbin R. Fast and accurate long-read alignment with Burrows-Wheeler transform. Bioinformatics. 2010. pmid:20080505.

2. Quinlan AR, Hall IM. BED Tools: A flexible suite of utilities for comparing genomic features. Bioinformatics. 2010. pmid:20110278.

**Supplementary Method Table SM1 Data preprocessing software and main parameters**

| **Software-1** | **get_umhost_IC_qc** |
| --- | --- |
| Version No. | v 2.0 |
| Specific parameters | The parameters are fixed in the software, not optional |
| Main purpose | 1. Eliminate reads containing low-quality bases with a proportion greater than the threshold (average Phred quality score <20) 2. Eliminate reads containing N bases greater than the threshold (>10%) 3. Eliminate reads containing linker contamination 4. Eliminate low complexity reads |
| **Software-2** | **PRINSEQ-lite** |
| Version No. | v 0.20.4 |
| Specific parameters | -derep -derep_min -lc_method -lc_threshold |
| Main purpose | Remove low-complexity sequences and repetitive sequences |
| **Software-3** | **samtools** |
| Version No. | v 1.9 |
| Specific parameters | rmdup -s |
| Main purpose | Remove repetitive sequences |

**Supplementary Method Table SM2 Database comparison software and main process**

| Host genome alignment software | Burrows-Wheeler Alignment (BWA) |
| --- | --- |
| Version No. | 0.7.17-r1188 |
| Sequence alignment parameters | mem -k 19 -t 8 -Y |
| Comparison result filter threshold | Read alignment ratio >= 0.5 to filter |
| Host Genome Database Sources | NCBI Refseq |
| Host genome database construction method | GRCh38 |
| Microbial Genome Alignment Software | BWA |
| Version No. | 0.7.17-r1188 |
| Specific parameters | mem -k 19 -t 8 -Y -h 10000 |
| Comparison result filter threshold | Read alignment rate < 0.8 or base mismatch rate > 0.1 to filter |
| Microbial Genome Database Source | NCBI Refseq, Genebank, Eupathdb |
| Microbial genome database construction method | Species strain genomes were clustered and screened for representative sequences |

**Supplementary Method Figure SM1 Overall interpretation fowchart**


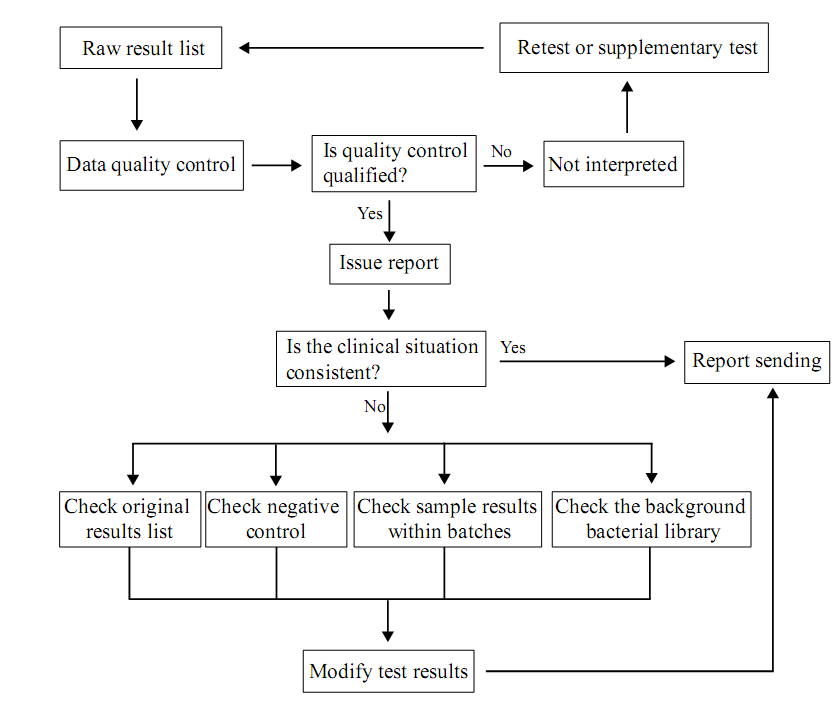

Supplement: Supplementary file 1 [file Table_1.docx]
