# Supplementary material for: Comprehensive evaluation of plasma microbial cell-free DNA sequencing for predicting bloodstream and local infections in clinical practice: a multicenter retrospective study
Source: Front Cell Infect Microbiol. 2024 Jan 4;13:1256099. doi: 10.3389/fcimb.2023.1256099 (PMC10868388; doi:10.3389/fcimb.2023.1256099)
Supplement: Supplementary file 2 [file Table_2.docx]

**Supplementary file 2**

**Supplementary Tables**

Table S1: Comprehensive Clinical Criteria for Determining Microbial Infection

Criteria Type of Evidence

| Proven | Having a clinical picture consistent with microbial infection with one of the following  - Presence of histopathologic, cytopathologic, or direct microscopic examination consistent with microbial for a specimen that was collected from a sterile site  - Presence of positive culture from sterile sites  - Presence of positive pathogenic microbial from normal bacterial parts  - True positive result based on Table 2 criteria for plasma mNGS-based detection |
| --- | --- |
| Probable | - Infection-related clinical laboratory tests such as procalcitonin, C-reactive protein, neutrophils, interleukin-6, beta glucan(1-3)-D test(G test) and serum galactomannan test(GM test), PCR  - Infection-related imaging manifestations  - Presence of infection-related clinical critera  - Presence of infection-related symptoms  - Consistent with confirmed positive or other conditions not confirmed positive but consistent with infectious diagnosis based on Table 2 criteria for plasma mNGS-based detection |
| Possible | - Presence of microbial infection history  - Physical examination suggests the possibility of infection  - False positive, true negative, or false negative result based on Table 2 criteria for plasma mNGS-based detection |

Note: The above criteria are intended to be used in combination with other clinical and laboratory findings, as well as the judgment of experienced clinicians.
